# Health Risks and Musculoskeletal Problems of Elite Mobile Esports Players: a Cross-Sectional Descriptive Study

**DOI:** 10.1186/s40798-022-00458-3

**Published:** 2022-05-13

**Authors:** Wing-Kai Lam, Rui-Tan Liu, Bob Chen, Xin-Zhou Huang, Jie Yi, Duo Wai-Chi Wong

**Affiliations:** 1 Sports Information and External Affairs Centre, Hong Kong Sports Institute, Hong Kong, China; 2 Dr. Chen Sport Training and Rehabilitation Research Center, Beijing, 101111 China; 3 Department of Biomedical Engineering, Faculty of Engineering, The Hong Kong Polytechnic University, Hong Kong, 999077 China

**Keywords:** Smartphone game, Sports injury, Electronic sports, Ergonomic, Prolonged sitting

## Abstract

**Background:**

Mobile-gaming athletes sit in the same posture for prolonged periods, contributing to significant health risks. This study investigated the health profiles, fatigue, pain and complaints, and musculoskeletal problems of full-time mobile-gaming athletes.

**Methods:**

A total of 50 elite mobile-gaming athletes were involved in this study. They were the starting lineup players from all ten professional teams competing in a top-tier multiplayer online battle arena tournament. A survey was conducted to evaluate their fatigue patterns, pain levels, and complaints. A descriptive analysis was conducted to evaluate the athletes’ health profiles [body mass index (BMI), fat ratio], fatigue, number of complaints, and musculoskeletal problems. The associations of career duration with BMI, fat ratio, and the total number of confirmed injuries were then determined using Spearman’s rank correlation test.

**Results:**

A total of 46% and 44% of the participants felt tired frequently and occasionally, while 34% and 58% experienced eyestrain frequently and occasionally, respectively. More than 30% of the participants reported headache and rhinitis. A longer esports career duration was associated with a reduction in BMI (*r* = −0.272, *p* = 0.056). Career duration had no significant association with smoking habits (*p* = 0.666), alcohol habits (*p* = 0.655), coffee habits (*p* = 0.946), rounds of games for which the player could maintain concentration (*p* = 0.253), ease of eyestrain (*p* = 0.569), tiredness (*p* = 0.510), dizziness (*p* = 0.071), or leg numbness (*p* = 0.318).

**Conclusion:**

The findings of this study stress the significance of esports injuries and indicate preventive measures for both athletes and recreational players.

## Key Points

- More than 90% of the mobile-esports athletes felt tired and experienced eyestrain.
- More than 30% of the mobile-esports athletes reported headache and rhinitis.
- Esports career duration was not associated with BMI or fat ratio.
- Esports career duration was not associated with smoking, drinking, or coffee habits.

## Introduction

Electronic sports (esports) is a form of competition in which players or teams battle in a virtual multiplayer video game through electronic interfaces (computer or mobile phone), particularly in a professional context. Esports have been accepted as an official medal event for the Asian Games and Olympic Games in 2022 and 2024, respectively [1]. In 2021, it was estimated that there were more than 2.6 million mobile gamers in the world and that nearly half of them came from Asia [2]. In China, approximately 560 million people, equivalent to 70% of the country’s online population, have played computer and mobile games regularly, accounting for US$11.1 billion in esports revenue streams in 2016 [3]. Previous research estimated a total global revenue of US$152 billion in 2019 and forecasted a revenue of over US$257 billion for the esports industry by 2025 [4]. However, it is difficult to estimate the number of esports athletes because there is no clear definition of “professional players” across studies and the esports community [5, 6]. In fact, amateurs regard players as playing “competitively” or “professionally” in online tournaments [6]. The multiplayer online battle arena (MOBA) is a popular type of online game worldwide. League of Legends and DotA 2 are the most played MOBA games, and the League of Legends championships sold out and attracted more than 27 million online participants [7]. MOBA games are similar in concept but differ in characters, executions, and stories. Typically, two teams of five players, with each player controlling a single character, compete against each other [7].

Unlike traditional sports that promote physical fitness, full-time athletes sit 5.5 h to more than 10 h a day during their regular game training to develop superior eye–hand coordination, fast reaction time, and rapid decision-making [4, 8–10]. The high training intensity with a specific postural behavior and work environment may induce health risks similar to sedentary lifestyles [9, 11]. Previous studies have reported that 6–8 h of sitting may increase all-cause mortality, cardiovascular disease mortality [12], and orthopedic injuries due to poor posture [5, 8, 13].

Esports injury and health investigations have focused on computer-based esports, but research on mobile esports games is lacking [4, 8]. A recent report indicated that more than 90% of millennials prefer to use their mobile devices instead of computer-based devices to play games online [14]. Mobile gamers have different playing postures, play in different environments, and have different player–environment interactions than computer-based gamers owing to the differences in screen size, viewing angle, and finger, hand, and arm motions. Therefore, they may have different postural adaptations, muscle fatigue/strain, and repetitive injury patterns after prolonged and high-intensity training.

Professional esports athletes stay seated and stare at screens, and excessive exposure to the blue-light spectrum from the screen can lead to eye fatigue and cause retinal and photoreceptor damage [15]. Prolonged occupational sitting time was found to be associated with chronic diseases and premature death [16, 17]. The associated weight gain from prolonged sitting and sedentary behavior among esports players could also be related to the risk of cardiovascular disease and diabetes [18, 19]. A prolonged sitting posture and unhealthy lifestyles (smoking, drinking, and caffeine intake) might interact and exaggerate musculoskeletal problems and health risks [20], and these unhealthy lifestyles/habits have been associated with playing video games [21]. In addition to these health risks, prolonged mobile-gaming postures (forward head, slouched posture, and rounded shoulder) impose problems for the musculoskeletal system [22–24]. Physical ailments in the trunk, upper limbs, and lower limbs, such as neck (42%), back (42%), wrist (36%), and hand/finger (32%) tendinopathy, have been reported [4, 5, 8]. In fact, the neck and back muscles are activated in an abnormal posture/position during prolonged sitting, which can result in muscle strain and fatigue. Fatigued muscles weaken the proper function of spinal support and increase mechanical stress on the ligaments and intervertebral discs, causing musculoskeletal pain and discomfort [25]. Repeated or sustained action, non-neutral posture, and repetitive forceful motion are also risk factors for muscle imbalance as well as strain/tendinopathy [5]. Mobile-esports athletes face heavy and unique physical demands in their “occupation,” which are associated with musculoskeletal problems [5]. More research is needed to facilitate best-practice guidelines for musculoskeletal health care in esports, especially with the remarkable growth of the industry [5].

In particular, the health risks of high-intensity mobile gaming or mobile phone usage are potential public health burdens that may not be confined to professional players. With the popularity of high-performance mobile phones, mobile games, and speedy 5G networks, adolescents and adults spend their days on mobile phones for communication, entertainment, and work, with a high incidence of related orthopedic injuries [26, 27]. An investigation of esports competitors could provide insights into the underlying health or musculoskeletal problems that provide further insights into the education on and prevention of injuries. Hence, the objective of this study was to understand health risks and complaints among full-time elite mobile-esports players.

## Methods

### Participants

The study included 50 male elite mobile-gaming athletes who were starting lineup players from ten professional teams of a top-tier MOBA tournament (Onmyoji Arena Pro League, OPL), which was hosted in the spring of 2021 in Shanghai. The tournament was a ten-team tournament with a 5-on-5 game competition. Onmyoji Arena was considered one of the best MOBA games for Android applications in 2022 [28]. Similar to other common MOBA games (e.g., League of Legends and DotA 2), each team player in Onmyoji Arena controls a unique character with a specific role and the power to collaborate with each team player to compete against opponent teams. To win a match, a team is required to win three out of five games or five out of seven games in the regular and playoff seasons, respectively, and each game lasts 15–20 min (www.onmyojiareana.us), which is comparable to the average match time in League of Legends (25–55 min) and DotA 2 (40 min) [29]. The study was approved by the institutional review board. All participants signed an informed consent form prior to the study.

### Experimental Protocol

The study was conducted during the off-season period (i.e., September 2021). Basic information, including body weight, height, and career duration in professional tournaments, was collected from the participants, as shown in Table 1. The participants were also asked if they had smoking, drinking, or coffee habits. Fat ratio (body fat percentage) was measured with the participants wearing thin/short-sleeve clothing while standing upright and holding the handrails of a bioelectrical impedance body fat measurement device (body fat monitor HBF-302, OmRon, Japan).

**Table 1:** Demographic information (*N* = 50)

Variables	Value
Age, mean ± SD (years)	20.0 ± 1.67
Age, range (years)	18–24
Body height, mean ± SD (m)	1.76 ± 0.06
Body weight, mean ± SD (kg)	70.82 ± 17.6
BMI, mean ± SD (kg/m^2^)	22.86 ± 5.11
Fat ratio, mean ± SD (%)	18.69 ± 8.10
Career duration, median (months)	6
Career duration, IQR (months)	3–11
Individuals with habits (*n*, %):	
Smoking	11, 22%
Alcohol	19, 38%
Coffee	28, 56%

SD, standard deviation; m, meter; kg, kilogram; BMI, body mass index; IQR, interquartile range

#### Questionnaire

The questionnaire included two parts and was written in Chinese. In the first part, the participants were given a list of symptoms/health problems, including eyestrain/dry eyes, headache/dizziness, somnipathy, anxiety neurosis, sleep disturbance, shoulder pain, scapula pain (periscapular pain), lumbar muscle strain, hemorrhoids, varicose veins, stomach pain/upset, angina pectoris, loss of appetite, and rhinitis. The list was selected on the basis of previous studies on mobile gaming [22–24] and those related to a sedentary lifestyle [9, 11, 12]. The participants were asked if they had experienced any of the listed health problems in the past 6 months and were instructed to indicate which ones. In addition, a body diagram was presented to the participants in the questionnaire. They were required to circle the body location where they felt pain or discomfort. For each circled location, they rated their level of pain or discomfort on a 10-point Likert scale, in which 0 points indicated no pain, 1–3 points indicated mild pain, 4–6 points indicated normal functioning, and 7–9 points indicated severe pain for which they had sought medication [30]. A physiotherapist was on stand-by to answer any queries about the list and the questionnaire.

In the second part, we adopted a supervised survey approach to assess the fatigue features of the participants [31]. The survey questions consisted of seven items with three options each (Table 2).

**Table 2:** Items in the supervised questionnaire

Items	Questions	Options
a	How many sets of game competitions at most can you maintain concentration?	1 set
		2 set
		3 set
b	When do you easily feel tired in the day?	Morning
		Afternoon
		Evening
		Midnight
c	How often do you get tired after prolonged gameplay?	Never
		Occasionally
		Frequently
d	How often do you get eyestrain after prolonged gameplay?	Never
		Occasionally
		Frequently
e	How often do you feel dizziness (i.e., feel like you have a lack of blood supply to the brain) after prolonged gameplay?	Never
		Occasionally
		Frequently
f	How long does it take to have leg numbness in a training or competition?	Never
		About 4 h
		About 8 h
g	Will you decide to relax after training?	Never
		Occasionally
		Frequently

### Data Analysis

A descriptive analysis was conducted to evaluate the health profile (BMI and fat ratio), fatigue features, number of self-reported complaints, and musculoskeletal problems among the participants. For musculoskeletal problems, the level of pain was also presented. The associations of career duration (in months) with BMI, fat ratio, and the total number of body regions with problems were determined using Spearman’s rank correlation test to assess the dose–response effect on the risk of health problems and weight gain (and thus the risks of related chronic problems).

Afterward, we categorized the participants into three groups based on their career duration: (1) < 6 months, (2) 7–12 months, and (3) over 12 months. The associations between the categorized career duration, habits (smoking, alcohol consumption, and coffee consumption), and selected fatigue features, including concentration tolerance, ease of eyestrain, tiredness, dizziness, and leg numbness, were evaluated using the chi-squared test. If the data assumption for chi-squared was violated, the likelihood ratio was implemented. The significance level was set at *p* < 0.05 for all analyses. All statistical analyses were performed using SPSS 26.0 software (IBM, Armonk, NY, USA).

## Results

### Descriptive Analysis

**Fig. 1:**
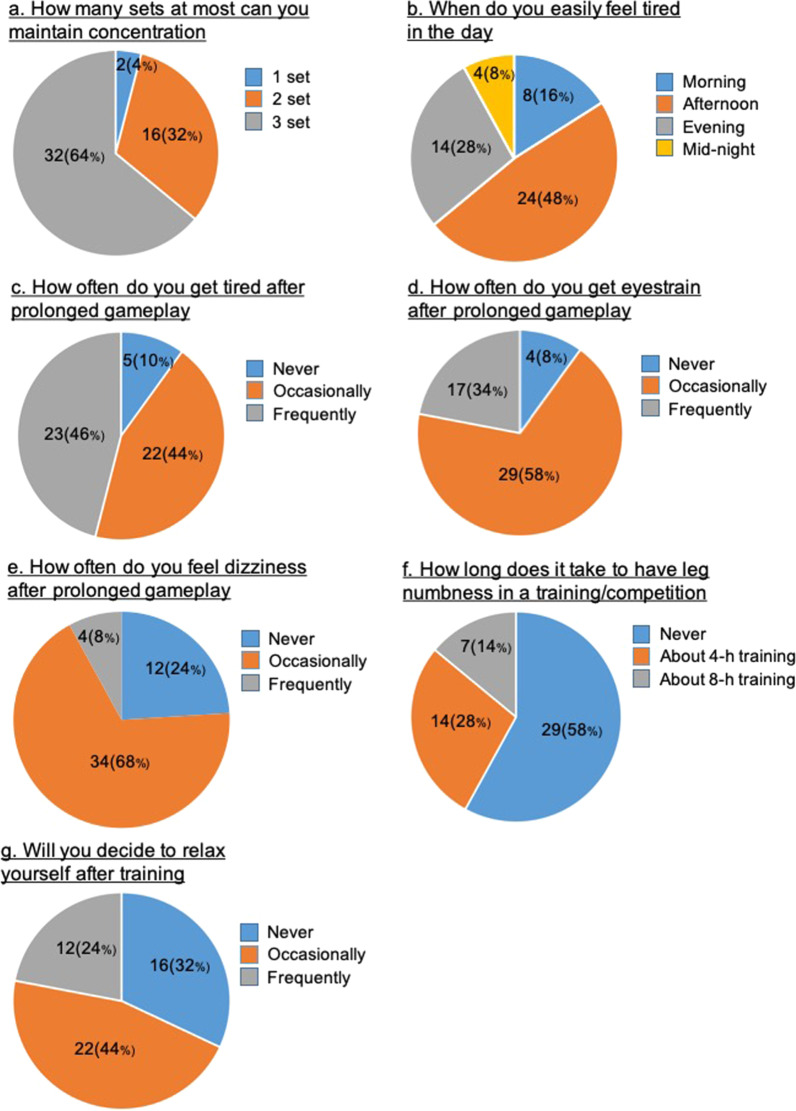
Reported fatigue characteristics in training and competition

For the measurement of fatigue in the supervised survey, 64% (*n* = 32) and 32% (*n* = 16) of elite athletes could maintain attentional concentration for two and three sets of a game, respectively (Fig. 1a). The onset of tiredness usually occurred in the afternoon (48%, *n* = 24) and evening (28%, *n* = 14) (Fig. 1b). In addition, 46% (*n* = 23) and 34% (*n* = 17) of the athletes felt tiredness and eyestrain frequently, while 44% (*n* = 22) and 58% (*n* = 27) of them felt tiredness and eyestrain occasionally (Fig. 1c and d), respectively. Eight percent (*n* = 4) of the athletes reported frequent dizziness, and 68% (*n* = 34) of them had occasional dizziness (Fig. 1e). A total of 28% (*n* = 14) of the participants experienced leg numbness after 4 h of training (Fig. 1f). A total of 24% (*n* = 12) and 44% (*n* = 22) of the athletes occasionally and frequently performed relaxation after training or competition, respectively (Fig. 1g).

**Fig. 2:**
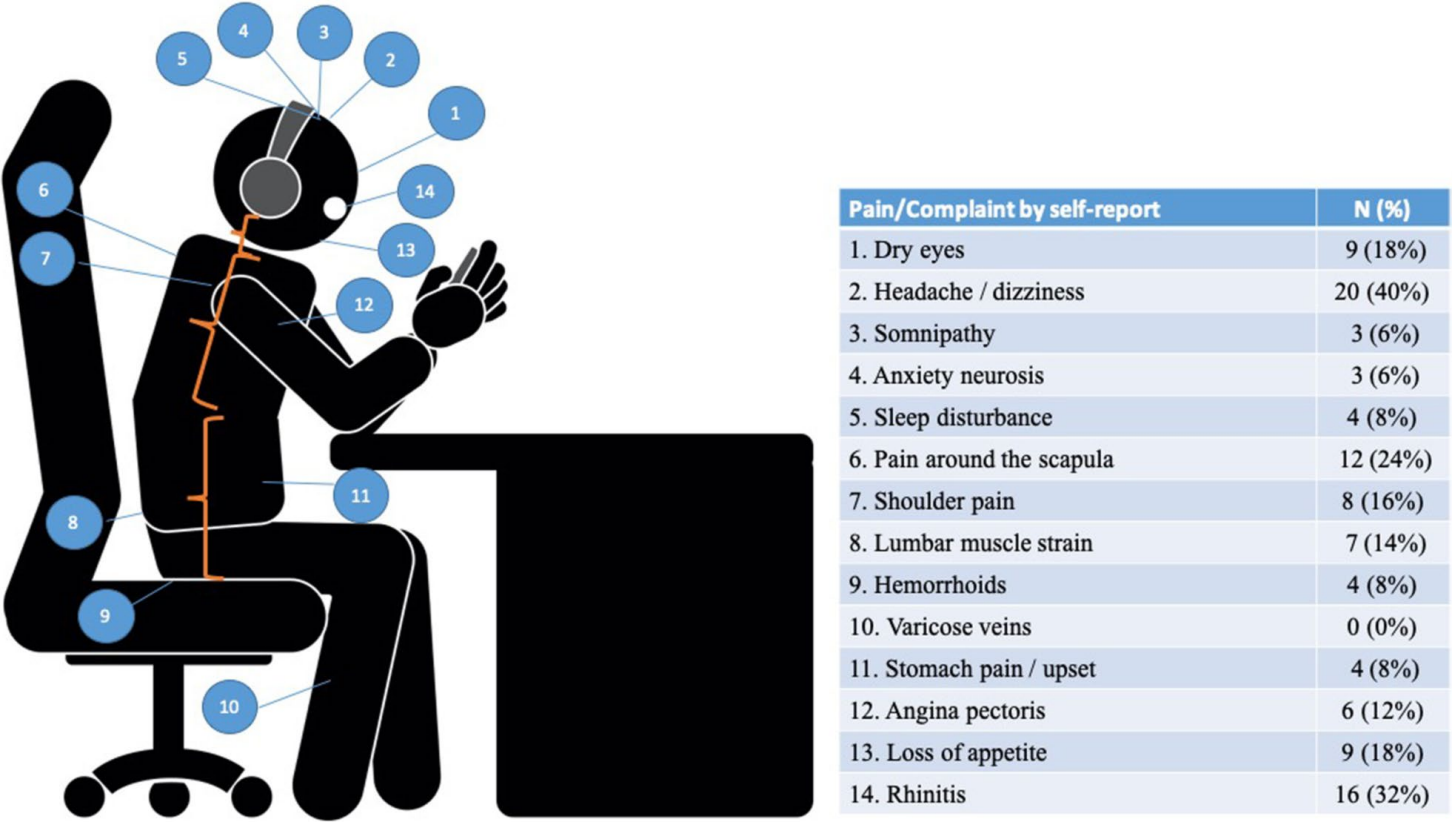
Pain/complaint location and frequency

Figure 2 shows a list of complaints reported by the participants. Headache/dizziness (40%, *n* = 20), rhinitis (32%, *n* = 16), and pain around the scapula (24%, *n* = 12) were the most common complaints.

As shown in Table 3, for the assessment by body region, the neck (40%, *n* = 20) was the most prevalent site of complaint, followed by the finger (38%, *n* = 19) and the head (32%, *n* = 16). A total of 20% (*n* = 10) and 24% (*n* = 12) of the participants suffered pain in the lower back and upper back, respectively. Although the majority of the pain was mild (91 out of 101 cases), there were four cases of moderate finger pain and three cases of moderate headache.

**Table 3:** Level of pain of specific regions graded by a 10-point Likert scale (nil 0, mild 1–3, moderate 4–6, severe 7–9)

Region	Case	(%)	Mild				Moderate				Severe			
			1	2	3	Total	4	5	6	Total	7	8	9	Total
Head	16	(32%)	–	7	6	13	2	1	–	3	–	–	–	0
Eye	9	(18%)	1	8	–	9	–	–	–	0	–	–	–	0
Neck	20	(40%)	1	11	8	20	–	–	–	0	–	–	–	0
Shoulder	8	(16%)	2	5	–	7	1	–	–	1	–	–	–	0
Upper back	12	(24%)	–	3	9	12	–	–	–	0	–	–	–	0
Finger	19	(38%)	1	12	2	15	4	–	–	4	–	–	–	0
Wrist	4	(8%)	–	2	2	4	–	–	–	0	–	–	–	0
Lower back	10	(20%)	1	2	5	8	2	–	–	2	–	–	–	0
Knee	3	(6%)	–	2	1	3	–	–	–	0	–	–	–	0

### Test for Association

Career duration had no significant association with BMI (*r* = −0.272, *p* = 0.056), fat ratio (*r* = −0.229, *p* = 0.110), or total number of body regions with health problems (*r* = −0.155, *p* = 0.284). In addition, career duration did not demonstrate a significant association with smoking habits (*p* = 0.666), alcohol habits (*p* = 0.655), coffee habits (*p* = 0.946), rounds of games for which the player could maintain concentration (*p* = 0.253), ease of eyestrain (*p* = 0.569), tiredness (*p* = 0.510), dizziness (*p* = 0.071), or leg numbness (*p* = 0.318) on the likelihood ratio tests.

## Discussion

Mobile gaming has become an increasingly popular recreational activity as well as an Olympic activity, with remarkable popularity worldwide [4, 8, 19]. However, a number of occupational factors, including prolonged screen time, repetitive movements, poor posture, and sedentary behavior, have been identified to increase the potential risk for the development of chronic diseases and possibly all-cause mortality [12, 32]. This study aimed to examine training behavior, fatigue profiles, and location of pain/discomfort among professional mobile-gaming athletes. The present findings reveal that most professional esports athletes experience physical fatigue and dry eyes after half-day (4 h) and full-day (8 h) training. Furthermore, similar to previous demographic studies, the study shows that esports athletes are required to sit in the same position for 5.5–10 h per day [8, 9] or up to 14 h per day [4], leading to a sedentary lifestyle with an increased chance of fatigue and chronic injury as well as increased all-cause mortality [12, 32]. Physical fatigue may be due to poor workplace practices (prolonged posture, repetitive movement), competition stress, and burnout, while eye fatigue could also be related to prolonged visual attention to the screen and a poorly lit environment [8].

Fatigue is a common symptom of many medical conditions, from mild to severe. It can cause various physical, mental, and emotional symptoms, including headaches/dizziness, sore muscles, muscle imbalance, poor concentration, impaired decision-making, and poor hand-to-eye coordination [33]. Our data indicate that nearly half of the athletes actively performed regular relaxation/recovery after esports training and competition, which agrees with a previous study showing that 29.2% and 34.2% of video-gaming athletes had relaxation/recovery and physical fitness sessions, respectively [19]. Therefore, regular fatigue monitoring and management sessions are recommended to maintain athletes’ best physical performance and reduce their injury and illness risk, especially after half-day and full-day training.

The prevalence of chronic musculoskeletal pain among general mobile-phone users was found to range from 4.2% to 13.3% [34], while a review reported a range from 8.2% to 89.9% depending on age, occupation, and the examined conditions [35]. Our study found that over 30% of the elite esports athletes reported headache/dizziness and rhinitis. More than two-thirds of regular mobile users reported musculoskeletal pain, including head, neck, shoulder, upper extremity, and back pain [36, 37], while the frequency of mobile gaming was associated with shoulder pain [38]. The differences could be related to the specific ergonomic and activity demands. In typical mobile-gaming athletes, maintaining posture with a prolonged inferior viewing angle and hand-held mobile-gaming position could induce head flexion, repeated or sustained wrist bending, and repeated twisting or pushing thumb motions [31]. Athletes may develop muscle imbalance between agonist and antagonist muscles and, thus, poor balance and muscle soreness. Additionally, our results indicate that the highest prevalence rates of symptoms among professional mobile-gaming athletes were for neck (40%), finger (38%), and head (32%) pain, which were slightly different from those of video-gaming athletes [neck (42%), back (42%), wrist (36%), and hand (32%)] [8] and those of sedentary office workers [lower back (72%) and neck (55.2%)] [39]. The differences in prevalence rates and locations of injuries could be due to the unique workplace environment and task intensity in mobile esports, which affect the fine-tuning of postural control and postural adaptation [40] and thereby lead to the development of chronic injuries [4, 5]. Another plausible explanation could be the different postures and muscles (thumbs) used in mobile gaming than in computer gaming with a keyboard/mouse and monitor. Clinically, prolonged mobile phone use/gaming with a more flexed neck position is linked to neck, shoulder, and upper limb pain [23, 41], and repetitive movements of the thumbs/fingers can lead to thumb/finger tendinopathy due to the increased use of the thumbs/fingers to push and twist during games [42].

Interestingly, our results indicate that career duration had no significant association with the fat ratio and total confirmed injury, which did not support our original hypothesis. It is expected that prolonged sitting time in mobile-game training could be similar to sedentary lifestyles [9], which could lead to physical inactivity that is related to an increased BMI and fat ratio and health risks such as hypertension and diabetes. One plausible explanation is the relatively short careers of professional athletes, who usually start at 18 years old and normally retire at 24 years old. Young sport athletes could be more likely to attain superior eye–hand coordination (500–600 action moves/min) and rapid decision-making and reaction times [8, 10]. Another explanation is that the movement intensity of esports is not as high as that of combat sports and might lead to only mild injuries, which may not easily recur over the years as in other traditional sports, such as basketball, soccer, and badminton. To the best of our knowledge, this is the first work that shows the fatigue and pain profiles of professional mobile-gaming athletes.

Some limitations should be considered in the interpretation of our results. First, only a single group of elite male mobile-gaming athletes from mainland China’s top teams was recruited in this study. The results may not be generalized to lower-level game players or recreational mobile gamers. Second, only self-report on health complaints was provided. Other comprehensive physiological and performance indicators (e.g., eye–hand coordination, decision-making capacity, visual attention) should also be considered to facilitate the identification of talented players. Third, we did not report the detailed daily activities of each participant in terms of their gaming, training, and relaxation regimes, which are considered contributing factors to the development of musculoskeletal pain. In the future, applying computer vision and machine learning approaches can help to track physical activities to provide concise information to develop training and recovery regimes. Fourth, fatigue and pain profiles are rather game-content specific, which may affect the decision-making and motor skill control (thumb versus index finger) to attain the best results in a game. Although all the participants were trained with the same game content in this season, more game content should also be investigated to determine how game content influences health injury. This can serve as the game design guideline (e.g., length of each game set, use of control setting) for game designers and policymakers.

## Conclusion

We found a high prevalence of headache, neck pain, and finger pain and a high occurrence of head and trunk injuries among elite mobile-gaming athletes, although no significant association was found with career duration. Future studies should consider investigating the influence of different intensities of and exposures to mobile games and direct preventive measures for game players.

## Data Availability

The datasets generated and/or analyzed in this study are available from the corresponding author on reasonable request.
